# Injury Severity and Contributing Driver Actions in Passenger Vehicle–Truck Collisions

**DOI:** 10.3390/ijerph16193542

**Published:** 2019-09-22

**Authors:** Jingjing Xu, Behram Wali, Xiaobing Li, Jiaqi Yang

**Affiliations:** 1 School of Transportation, Wuhan University of Technology, Wuhan 430063, China; xjj087@gmail.com (J.X.); jackyyoung@126.com (J.Y.); 2 Senseable City Lab, Massachusetts Institute of Technology, Cambridge, MA 02139, USA; bwali@mit.edu; 3 Alabama Transportation Institute, University of Alabama, Tuscaloosa, AL 35487, USA

**Keywords:** passenger vehicle–truck, injury severity, unsafe driver actions, random parameters, ordered probit, freight transportation

## Abstract

Large-scale truck-involved crashes attract great attention due to their increasingly severe injuries. The majority of those crashes are passenger vehicle–truck collisions. This study intends to investigate the critical relationship between truck/passenger vehicle driver’s intentional or unintentional actions and the associated injury severity in passenger vehicle–truck crashes. A random-parameter model was developed to estimate the complicated associations between the risk factors and injury severity by using a comprehensive Virginia crash dataset. The model explored the unobserved heterogeneity while controlling for the driver, vehicle, and roadway factors. Compared with truck passengers, occupants in passenger vehicles are six times and ten times more likely to suffer minor injuries and serious/fatal injuries, respectively. Importantly, regardless of whether passenger vehicle drivers undertook intentional or unintentional actions, the crashes are more likely to associate with more severe injury outcomes. In addition, crashes occurring late at night and in early mornings are often correlated with more severe injuries. Such associations between explanatory factors and injury severity are found to vary across the passenger vehicle–truck crashes, and such significant variations of estimated parameters further confirmed the validity of applying the random-parameter model. More implications based on the results and suggestions in terms of safe driving are discussed.

## 1. Introduction

The economic impacts and safety hazards resulting from truck-involved crashes highlight freight transportation safety as a contemplative societal concern [1,2]. From 2014 to 2015, the vehicle miles traveled by large trucks (gross vehicle weight > 10,000 pounds) increased by 0.3%, while the number of fatal large truck-involved crashes increased by 8% [3]. In addition, truck-involved collisions are often more disruptive (e.g., damage to vehicles, roadway systems, as well as other facilities) and costly (e.g., loss of life, use of emergency medical service (EMS), property damage, and traffic congestion) [4]. From an injury severity perspective, in 2014, a total of 3600 people died in large truck-involved crashes, out of which 16% were truck occupants and 68% were occupants of passenger vehicles [5].

Although most of the truck-involved crashes involve multiple vehicles, two-vehicle passenger vehicle–truck collisions often contribute to the majority (about 48%) of total fatal crashes according to the 2017 Fatality Analysis Reporting System (FARS) on fatal truck-involved crashes. For instance, in 2014, two-thirds of all police-reported crashes involved a truck and another vehicle [6], and 63% of the fatal large truck crashes involved two vehicles [6]. Alarmingly, 97% of the vehicle occupants who died in two-vehicle passenger vehicle–truck crashes were the occupants of passenger vehicles [5]. Therefore, this study focuses on crashes with one passenger vehicle and one truck. Another reason why this paper focuses on two-vehicle passenger vehicle–truck crashes is the lack of clarity in identifying the drivers who acted unsafely. Despite significant improvements in decreasing the national toll of truck-involved fatalities, based on the statistics there is still an urgent need for careful investigation of the factors that are associated with injury severity outcomes in two-vehicle passenger vehicle–truck collisions.

Some studies have successfully disentangled the complex associations between key factors (such as driver age, gender, alcohol intake, and over-speeding) and the most severe injury outcomes in truck-involved collisions [7,8,9,10]. However, questions concerning which unsafe driver (truck or passenger vehicle driver) with intentional or unintentional improper driving actions will result in the most severe injury in a crash level have been under-researched in the literature; in particular, among the unsafe driving actions, which ones can substantially contribute to severe injury outcomes [11,12,13]. In addition, due to several unobserved crash-, vehicle-, and driver-related factors, injury severity models often do not show well goodness-of-fit if the models do not account for unobserved heterogeneity. Ultimately, the modeling results are biased and can adversely affect the policy implications of risk factors or countermeasures. Thus, the present study addresses the methodological concern of unobserved heterogeneity by capturing the complexities embedded in the data of passenger vehicle-truck collisions. This is achieved by estimating random-parameter ordered probit models and comparing with traditional fixed-parameter ordered probit models. 

The present study contributes by focusing on two objectives:

1. Quantify the associations between unsafe driving behaviors and injury severity outcomes in passenger vehicle–truck collisions. Specifically, the intentional and unintentional driving actions of truck and passenger vehicle drivers.
2. Explore unobserved heterogeneity in the associations of injury severity with unsafe pre-crash behaviors, while controlling for the driver, vehicle, and roadway factors.

An explicit investigation of drivers’ unsafe pre-crash actions is likely to allow us to develop actionable safety improvement strategies. In order to achieve the objectives, sophisticated fixed- and random-parameter ordered probit models are estimated by using real-world police-reported crashes, which allows for the unearthing of important and embedded relationships in crash data. The content of the paper is original and timely given the enormous costs sustained by society in consequence to passenger vehicle–truck collisions, and the implications of safety concerns for occupants of passenger vehicles. 

## 2. Literature Review 

Given the considerable costs imposed by truck-involved collisions, several researchers have investigated such collisions and the key factors that may be associated with injury outcomes. In this section, previous studies are synthesized with the specific focus on methodological approaches that are used for establishing associations between different crash characteristics, unsafe pre-crash behaviors, and injury outcomes in passenger vehicle–truck collisions. 

A broad spectrum of studies investigated the associations between several factors such as collision types, roadway types, vehicle types, and injury outcomes in passenger vehicle–truck collisions [4,14,15,16,17,18,19]. For instance, rear-end collisions, and right/left-turn crash types were found to be associated with higher injury severity [4,14,15]. From the perspective of the striking and struck vehicle, Duncan et al. [4] concluded a higher likelihood of severe injuries to passenger car occupants if struck by a truck [4]. From roadway related factors, studies by Lemp et al. [17] and Khattak et al. [16] concluded a higher likelihood of severe injury outcomes on curved sections and roadway sags [16,17]. Previous studies have shown that the relaxation of the speed limit often increases the number of fatalities, and the speed limit could reduce the possibility of accidents to a certain extent [20,21]. The lane width also has an effect on traffic safety. One study from Wu et al. concluded that the standard-sized lane (width around 3.45 m) is less likely to associate with a crash, while the undersized and oversized widths are often more likely to associate with crashes [22]. Regarding vehicle and driver-related factors, Chang and Mannering [15] and Christoforou et al. [18] found that a greater number of occupants in a vehicle (or weighted vehicles) is associated with higher injury outcomes [15,18]. Likewise, females, older people, and the non-use of seatbelts were found to be associated with higher injury outcomes [4,17,23]. Driver condition-related factors such as fatigue or falling asleep, driving under the influence, and physical or mental impairment are also documented to be associated with a higher possibility of injury severity [24,25,26].

The role of driver actions in truck-involved crashes has also received considerable attention [4,15,16,23,27]. Council et al. [28] investigated motor vehicle drivers’ unsafe driving actions (UDAs) that resulted in (or contributed to) passenger vehicle–truck crashes. The study concluded that most frequent unsafe behaviors were driving inattentively, improper merging, failure to stop or slow, and following too closely [28]. Likewise, several studies concluded that speeding was the riskiest driving behavior in truck-involved collisions [4,15,16,23]. 

The aforementioned studies did not focus primarily on investigating a broad range of unsafe pre-crash driving behaviors, intentional and unintentional improper actions of either truck driver or passenger vehicle driver, and the implications on most severe injury outcomes of passenger vehicle–truck collisions. An explicit investigation of drivers’ unsafe pre-crash actions can help in developing actionable safety improvement strategies for passenger vehicle–truck collisions. Furthermore, due to the complex crash data structure and different unobserved crash-, vehicle-, and driver-related factors, the associations between key driving behaviors and injury outcomes may vary significantly across different crashes. Ignoring the possibility of varying relations between key explanatory factors and injury outcomes can neglect important information embedded in passenger vehicle–truck crash data [29]. The study of Zou et al. [30] applied a generalized finite mixture of the negative binomial model with K mixture components to accommodate the heterogeneity. Xiong and Mannering [31] accounted for unobserved heterogeneity by combining the finite-mixture and random-parameter models. Finite-mixture and random parameter models are two main methods to examine the unobserved heterogeneity. Both studies above did not focus on passenger vehicle–truck collisions. Note that while the study by Islam and Hernandez [23] addresses unobserved heterogeneity in passenger vehicle–truck collisions using a unique national-level database, it did not focus explicitly on investigating intentional and unintentional pre-crash behaviors and their associations with most severe injury outcomes in such collisions. Regarding the effects of the heterogeneity, a copula regression model was used to identify the effect of underreporting on the analysis of wildlife-vehicle collisions by Zou et al. [32]. Ye and Lord [33] also identified the effects of underreporting on the models, which are commonly used in injury severity analysis. However, in this study, the random-effects ordered probit models were also estimated but did not result in significant improvement in the model fit compared with random-parameter models. We could perform a future study on other datasets to further explore the effects of the underreporting on different models.

## 3. Methodology

### 3.1. Data Source

**Figure 1:**
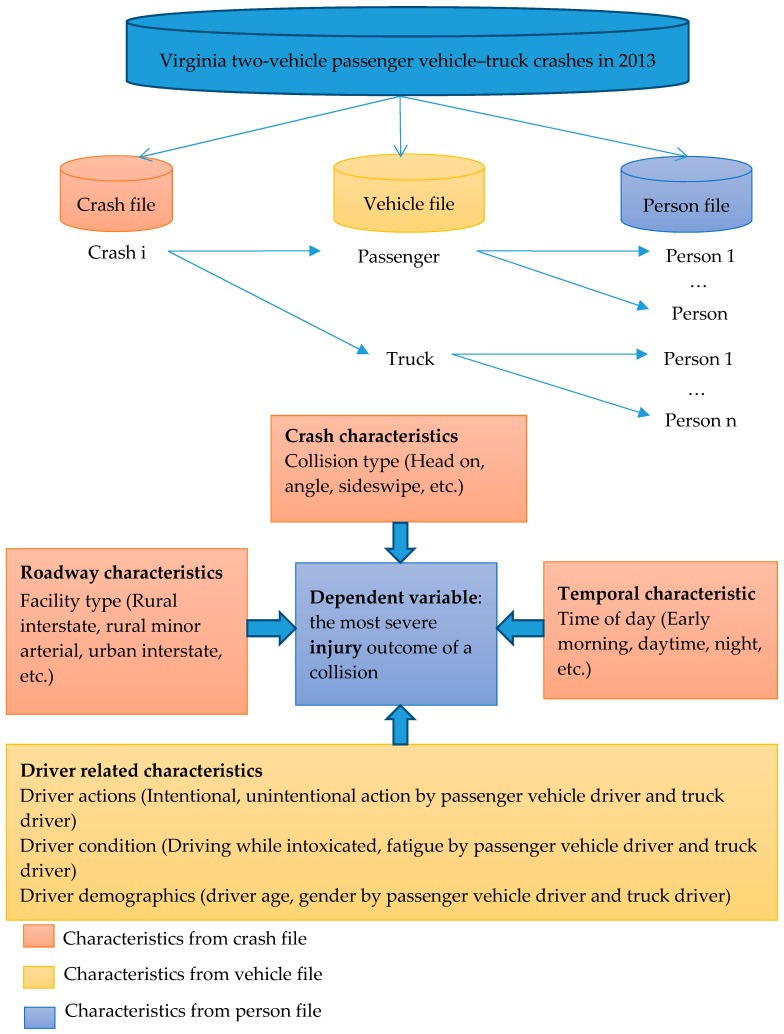
Data structure and conceptual framework.

This paper used the 2013 Virginia Police Crash Reports obtained from the Virginia Department of Transportation. The database is comprehensive and well-organized containing records of crashes occurring across Virginia. For this study, three files are extracted from the database and linked together in order to obtain the crash, vehicle, and person-level information involved in passenger vehicle–truck collisions. Specifically, the crash file contains information on variables describing the crash, crash time, roadway characteristics, and collision type—the vehicle file contains information on vehicles such as vehicle body type, and the person file contains information on occupants (including driver) such as age and gender, level of injury sustained, and other driver-related factors. All three files are linked together through a unique crash identification number. Figure 1 presents the data structure and the conceptual framework. Note that passenger vehicles in this study include passenger car, pick-up truck, van, and sports utility vehicles (SUV). 

Among 121,601 crashes documented in the database, 7501 are truck-involved crashes, and 4926 crashes are two-vehicle passenger vehicle–truck crashes. Given the focus of this study (as explained earlier), two-vehicle passenger vehicle–truck collisions are extracted (i.e., 4926), which accounts for a significant 66% of the total truck-involved collisions. Given the national average of 63%, the sample size at hand is reasonably representative. 

There are nine combinations of different (truck and passenger vehicle) driver actions that are shown in Table 1. Among them, the first four combinations account for around 90% of all combinations. Moreover, to better analyze the associations of drivers’ actions with most severe injury outcome, the cases in which only one driver (either passenger vehicle or truck) undertook an improper action are selected. From the collision type perspective, as the present study focuses on two-vehicle passenger vehicle–truck collisions, types such as non-collision, fixed object, collisions with train, motorcyclist, and animals are ignored. Finally, after data processing and cleaning, the resulting sample size contains 3774 passenger vehicle–truck collisions such as rear-end, angle, head-on, and sideswipe same and opposite directions.

In terms of injury severity, this study utilized the most severe injury among all the occupants in both vehicles as the injury severity level of the crash. As reported in the police crash report forms, five levels of injury severity are observed: killed, serious injury, minor injury, no apparent injury, and non-injury (property damage only). However, due to the limited number of crashes with fatalities, and in order to facilitate the analysis, the injury severity scale is categorized into fatal/serious injury, minor injury, and no injury. Several studies in the past have re-categorized injury severity scales due to limited cases with fatalities [34]. 

Finally, to analyze the associations between unintentional and intentional driver actions (either the truck driver or passenger vehicle driver), the driver actions reported in VDOT police crash report forms are classified into four categories:

Action 1: Passenger vehicle driver undertook no improper action while truck driver undertook an intentional improper action.

Action 2: Passenger vehicle driver undertook no improper action while truck driver undertook unintentional improper action.

Action 3: Passenger vehicle driver undertook intentional improper action while truck driver undertook no improper action.

Action 4: Passenger vehicle driver undertook unintentional improper action while truck driver undertook no improper action.

To have a general map of those actions, the intentional actions include behaviors such as speeding, following too close, wrong place or no right-of-way, disregard of officers, signals or signs, and so forth; the unintentional actions include driver behavior that related to driving technique inadequate, failing to maintain proper control, avoiding animals or object and other improper actions, and so forth. Note that the classification scheme adopted in this study is consistent with Liu et al. [35], who investigated pre-crash driver actions in work zones. For convenience, the four categories of unsafe pre-crash driver actions will be referred to as Actions 1 to 4 hereafter. 

### 3.2. Modeling Framework

An ordered probit modeling framework is used in this study due to the ordinal nature of the response outcome [16]. The model can be defined in terms of the ordinal dependent variable $Y^{*}$ as:

(1) $$Y^{*}=\beta X+\delta$$

where $Y^{*}$ is a dependent variable (in our case most severe injury outcome of a collision), β is a vector of estimated parameters, X is a vector of the explanatory variable (driver action, collision type, collision time, and etc.), and δ is error term, assuming it is normally distributed. Based on the ordered probit model with normal residual distribution and from Equation (1), the dependent variable $Y^{*}$ can be formulated as follows:

(2) $$Y\;={\;n\;\mathrm{if}\;\gamma }_{n-1}\leq Y^{*}<\gamma_{n}$$

where ${\;\gamma }_{n}$ denotes estimated parameters that define the observed ordinal data $Y^{*}.\;Y^{*}$ is related to the latent variable $Y^{*}$ through the estimated parameter $\gamma_{n}$. The probability of the ordered probit model as follows:

(3) $$P\left(y=n\right)=\emptyset \left(\gamma_{n}-\beta X\right)-\emptyset \left(\gamma_{n-1}-\beta X\right)$$

where ∅(.) is a function of normal cumulative distribution. 

Note that the above framework implies an unduly restrictive assumption of constant parameter effects across sampled observations. For instance, one coefficient is estimated for each explanatory factor at times when the associations between explanatory factors and injury severity may vary across sampled observations due to the presence of several observed and unobserved factors [34]. In the presence of such observed and unobserved factors (which are likely to be present in crash data), constraining the fixed model coefficients across observations could potentially result in biased parameter estimates, as shown in various studies [29,36]. Therefore, random parameters may be incorporated to solve this issue using new estimation procedures through simulated maximum likelihood estimation techniques as:

(4) $$\beta_{i}=\beta +\vartheta_{i}$$

In this study, the random-parameter ordered probit model is estimated by simulated maximum likelihood estimation, and by using 200 Halton draws as recommended by other studies [36]. For random parameters, we tested different distributions such as lognormal, triangular, Weibull, and normal distributions (discussed later). 

Finally, after model estimation, the signs of parameter estimates are of great importance. A positive sign shows an increase in the probability of the most severe injury severity and a decrease in the probability of the least severe outcome and vice versa for negative parameter estimates [29]. The coefficients can be also used to interpret the effects of explanatory factors on intermediate categories [16]. As such, marginal effects for sample means are estimated both for fixed and random-parameter ordered probit models as:

(5) $$\frac{\partial P\left(y=n\right)}{\partial X}=-\left[\emptyset \left(\gamma_{n}-\beta X\right)-\emptyset \left(\gamma_{n-1}-\beta X\right)\right]\beta^{\prime }$$

where $\beta^{\prime }$ is the impact of changes in X and ∅(.) is a function of standard normal distribution [34,37]. 

## 4. Results

### 4.1. Descriptive Statistics

**Figure 2:**
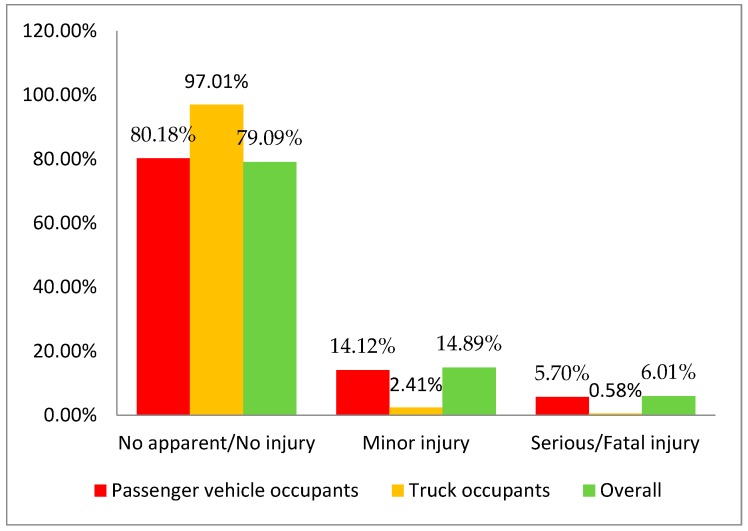
Most severe injury severity distributions in passenger vehicle–truck involved collisions.

The present study analyzes 3774 passenger vehicle–truck crashes, which involve 7548 vehicles and 7871 individuals. Figure 2 shows the distributions of most severe injury outcomes of the overall occupants in the collisions (green bars), most severe injury outcomes of the passenger vehicle occupants (red bars), and most severe injury outcomes of the truck occupants (orange bars). It shows the proportion of each injury category decreases as the injury severity increases. The majority of the crashes are no apparent/no injury collisions. The proportion of minor/possible injury is much less than the no apparent/no injury. The proportion of serious/fatal injury is the smallest, which only accounts for about 6% of the collisions. The distribution is consistent with the Federal Highway Administration (FHWA) highway safety statistics [38], which show a similar KABCO distribution (KABCO scale is a kind of classification of injury severity. K: fatal injury; A: incapacitating/suspected serious injury; B: non-incapacitating/suspected minor injury; C: possible injury; O: no/no apparent injury) indicating the scarce fraction of serious/fatal injury crashes among all crashes. The distributions provide important information embedded in the data. For instance, from an overall crash injury severity level distribution perspective, 6.01%, 14.89%, and 79.09% of the crashes were serious/fatal, minor, and no apparent/no injury, respectively. Importantly, the stratification of most severe injury outcomes based on passenger vehicle and truck reveals that, compared with truck occupants, passenger vehicle occupants are six times more likely to sustain minor injuries (14.12% vs. 2.41%) and ten times more likely to suffer serious/fatal injuries (5.70% vs. 0.58%). These findings are in agreement with several past studies, which confirm the reasonableness of the data structure [4,26,39,40]. 

Regarding the key explanatory variables, Table 2 presents descriptive statistics of variables included in the fixed- and random-parameter ordered probit models. Table 2 displays the mean, standard deviation (SD), and the variance inflation factors (VIFs) value for each indicator variable. Due to several unidentified interactions among key factors in crash data, multicollinearity may arise, which might significantly affect model results if not addressed carefully. The existence of multicollinearity among independent variables was checked by VIFs (Table 2). The VIF values of each variable are much smaller than 10, indicating the absence of significant multicollinearity [41].

Based on the descriptive statistics, the data seem to be of reasonable quality. In 37.5% of the collisions, passenger vehicle driver undertook no improper actions while truck driver undertook an intentional improper action (action 1), as opposed to 41.3% of collisions in which truck driver undertook no improper actions and passenger vehicle driver undertook intentional improper action (action 3). Almost 30% of passenger vehicle–truck collisions were angle collisions, and approximately 21% of them occurred between 01:00 and 08:00. The average total number of injured is 0.388 in all collisions (Table 2). 

### 4.2. Modeling Results

**Figure 3:**
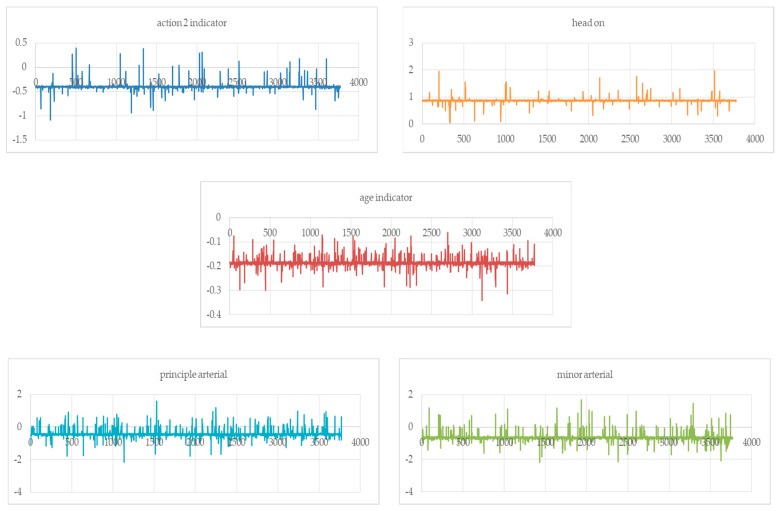
The variation of the random parameters.

Explanatory variables are identified by developing the simple correlation matrices of key factors with injury severity of the most severely injured person in passenger vehicle–truck collisions. This helped in identifying potential factors related to driver actions, collision types, roadway types, driver conditions, demographics, and temporal characteristics. Next, a series of fixed-parameter ordered probit models are estimated for injury severity in the collision. Most variables were either statistically significant at a 95% confidence level or theoretically important and retained for subsequent analyses. The results of the final fixed–parameter ordered probit model are presented in Table 3. Theoretically, fixed-parameter models constrain the parameter estimates to be fixed for explanatory variables across the entire sample of the passenger vehicle–truck collisions [29]. Given the fact that several observed and unobserved factors could contribute to injury severity in the collisions, random parameters are incorporated with respect to conventional (fixed-parameter) ordered probit framework. Conceptually, random-parameter models provide the flexibility to allow the parameter estimates to vary across sample observations with some pre-specified distribution [36]. The results of random-parameter ordered probit model are presented in Table 3. The final random-parameter model includes 15 correlates, of which five parameters exhibited statistically significant variability (as indicated by the standard deviation of parameter estimates) across the sampled collisions (Table 3). One of these parameters is related to truck drivers undertaking unintentional improper actions. Note that the variability exhibited by random parameters tends to be very large, indicating significant heterogeneity in the associations of these variables with injury outcomes. Figure 3 shows the variation of the coefficients. These results suggest the associations between some explanatory factors and injury outcomes are indeed varying across sampled collisions, with positive parameter estimates for some collisions and negative ones for others. Note that random-effects ordered probit models were also estimated but did not result in a significant improvement of the model’s goodness of fit compared with random-parameter models. 

The incorporation of random parameters resulted in the overall improvement of fitness compared with the fixed-parameter model (Table 3). Moreover, following the study of Washington et al. [42], a chi-square likelihood ratio test is conducted to investigate the statistical superiority of the random-parameter ordered probit model against its fixed counterpart. The likelihood ratio test statistic is LR = −2[LL(βa) − LL(βb)], where LL(βa) is the log–likelihood at the convergence of fixed-parameter (restricted ordered probit) model, and LL(βb) is the log-likelihood at the convergence of random-parameter (unrestricted ordered probit) model. The test statistic is χ² distribution is calculated based on certain degrees of freedom (i.e., the difference in numbers of parameters between fixed- and random-parameter models). With 22 degrees of freedom (i.e., five random parameters), the resulting χ² value is 42.780 (Table 3), which is very close to critical χ² 0.005 (99.5% level of confidence) of 42.8. As a consequence, a random-parameter ordered probit model is observed to provide statistically superior predicting capability against the fixed-parameter ordered probit counterpart [42]. Regarding the functional form of random parameters, normal, lognormal, and uniform distributions are tested (please refer to authors for more results). All normally distributed random parameters provided a better fit. This finding is in agreement with the traffic safety literature [34,36]. Finally, as discussed by Khattak and Rocha [43], in order to interpret the associations between explanatory factors and intermediate response category (minor injury), marginal effects are provided for fixed- and random-parameter models in Table 4. 

### 4.3. Discussion

To facilitate discussion of estimated models, the explanatory factors (Table 2) are categorized as driver actions, collision types, roadway types, driver-related factors, and time of day. Based off the modeling results, some variables (e.g., Action 2, principal arterial, and passenger vehicle driver age) are significantly associated with injury severity in the random-parameter model, but they are not as significant as in the fixed-parameter model. A possible explanation for this is the unobserved heterogeneity, which is one of the important contributions in this study. Note that some of the variables are not significantly based on their impacts on the injury outcome. Therefore, they are not included in the final model. The correlation estimations are substantially diversified among the fixed- and random-parameter models across the passenger vehicle–truck crashes as discussed below. Note that Figure 3 shows the variation of the random parameters.

#### 4.3.1. Driver Actions

Regarding driver actions (Table 3), action 2, 3, and 4 indicators reveal important associations between driver actions (for both truck and passenger vehicle driver) and injury outcomes. The action 1 indicator (if the passenger vehicle driver undertook no improper action while the truck driver undertook intentional improper action) is used as the base category. Action 3 and 4 indicators were statistically significant (at 95% and 99% level of confidence, respectively), while action 2 indicator is insignificant in the fixed-parameter model. Based on the random-parameter model, intentional improper action of the passenger vehicle driver (action 3), as well as the unintentional improper action of the passenger vehicle driver (action 4) are both significantly associated with higher injury outcomes. From a behavioral perspective, this finding is important in the sense that it highlights the higher propensity of suffering severe injuries given a crash, irrespective of passenger vehicle driver undertaking intentional or unintentional improper actions. Similar insights were observed in another study [23]. However, that study focused on maneuvers and not explicitly on intentional and unintentional actions [23]. Although action 3 and action 4 indicators are found to be fixed parameters, the incorporation of random parameters in the ordered probit framework significantly enhanced the statistical significance of estimated parameters. Passenger vehicle driver with no improper action and truck driver with unintentional improper action (action 2, normally randomly distributed) is statistically negatively significant associated with injury severity in the random-parameter model at a 99% level of confidence compared with action 1 (passenger vehicle driver undertook no improper action while truck driver undertook intentional improper action). A possible explanation is that the truck driver’s intentional actions, such as speeding or cutting in, are more likely to associate with higher injury outcomes than the unintentional actions of the truck driver. The truck drivers are often well trained so that they may be unlikely to make unintentional driver actions such as distraction (due to fatigue) or failing to maintain proper control. Thus, the unintentional action of the truck driver is negatively associated with injury severity compared to the action 1 base. The marginal effect of the action 2 shows that there is a 5% increase in the possibility of sustaining no apparent/no injury, a 4.9% decrease in the probability of suffering a minor injury, and a 0.2% decrease in suffering a serious/fatal injury (compared with the action 1).

#### 4.3.2. Driver Related Factors and Time of Day

Interestingly, if the passenger vehicle driver is fatigued or asleep in a collision, the injury severity is higher. Results show that fatigued driving is associated with injury severity at a 90% confidence level. While the parameter estimates for this variable are found to be fixed, the marginal effects obtained from fixed- and random-parameter models reveal differences. The average marginal effects for fatigue (Table 4) show that if the passenger vehicle driver is fatigued, then there is a 6.6% decrease in the probability of sustaining no apparent/no injury, a 6.3% increase in the probability of suffering a minor injury, and a 0.4% increase in probability of suffering a serious/fatal injury, all for the most severely injured occupants in a passenger vehicle–truck collision. Notably, truck driver fatigue did not have a statistically significant association with injury severity. This might be due to the fact that truck drivers are usually well trained to avoid fatigued driving. This result is interesting when coupled with the results that the passenger vehicle–truck collisions occurring late at night and in early morning hours (between 01:00 and 08:00) are more serious, which perhaps captures drivers’ drowsiness. The results show that late night/early morning indicator is positively associated with injury outcomes at a 99% confidence level in both models. The marginal effects in Table 4 show that compared with other times of day, a collision that occurs late at night and in early morning times has a 4.6% higher chance of suffering a minor injury, and a 0.2% increased chance of suffering a serious/fatal injury. Though late night/early morning indicator can be a fixed parameter, its t–value is improved in the random–parameter model. Drinking and driving by the passenger vehicle driver is associated with more severe injury outcomes at a 99% confidence level in both fixed and random models, which sounds logical. If the driver is fatigued or driving after drinking, he/she may be more likely to be involved in an accident. Moreover, such driving conditions are often correlated with severe injury severity.

In terms of driver demographics, the age of the passenger vehicle driver is found to be a normally distributed random parameter with a mean of −0.187 and a standard deviation of 0.232. Passenger vehicle drivers aged 20–29 years are negatively associated with injury outcomes. This finding is in agreement with other studies [43]. In this study, it is found that the driver in this age range has a 2.7% decreased likelihood of suffering a minor injury, and a 0.1% decreased probability of suffering a serious/fatal injury. One possible explanation for this is that the young drivers are physically strong, while the old drivers are often more vulnerable and more likely to be injured in a crash. Previous studies also show that young passenger vehicle drivers are less likely to sustain a severe injury than old drivers in more severe crashes, even though they are more likely to be involved in crashes. The results show that significance of age is found to be significantly enhanced in the random-parameter model. (Note that driver conditions and the demographics of truck drivers were also considered, but none of the variables were statistically significant, and thus the results are not presented.)

#### 4.3.3. Roadway Types

Compared with interstates, principal arterials, minor arterials, collector and local are all expected to be negatively associated with severe injury outcomes. Local roadway systems are more likely to be associated with less severe crashes, compared with other roadway types. Notably, principal arterials and minor arterials both resulted in parameter randomness. The significance of principal arterial and collector are improved from 95% in the fixed model to 99% in the random model. Principal arterials are normally distributed with a mean of −0.433 and a standard deviation of 0.849 (Table 3), suggesting a 6.1% increased probability of sustaining no apparent/no injury, a 5.9% decreased probability of sustaining a minor injury, and a 0.2% decreased probability of suffering a serious/fatal injury, compared with interstate. Likewise, heterogeneity is observed in associations between minor arterial and most severe injury outcomes (Table 3). Moreover, the marginal effects obtained from the fixed- and random-parameter models have significant differences, especially for the serious/fatal injury outcomes (Table 4). For collisions on the local roads, the chances of serious/fatal injury decrease by 0.7% (in the fixed-parameter model) as opposed to a 0.2% decrease in the random-parameter model. Even though other roadway types are at the same significance level in two models, the values of estimates are also improved in the random-parameter model. 

#### 4.3.4. Collision Types

Out of all collision-type factors, head-on and angle collisions are found to be statistically positively associated with the most severe injury outcomes in passenger vehicle–truck collisions, while sideswipe-same direction collisions are negatively associated with the injury severity. Note that both head-on and sideswipe-same direction collisions are significantly associated with injury severity at a 99% level of confidence in both models. The significance of angle collisions is found to be enhanced in the random-parameter model (95% and 99% level of confidence in the fixed model and random model, respectively). Numerous studies have found positive associations between collision types (head-on and angle) and injury outcomes. However, from a passenger vehicle–truck collision perspective, the injury outcomes may be more severe, which is potentially due to the enormous physical momentum of colliding trucks [4]. As such, the modeling results suggest a greater propensity of severe injury outcomes associated with both head-on and angle collisions. This finding is in agreement with Khattak and Targa [39] who investigated injury severity and total harm in truck-involved work zone crashes [39]. However, this study suggests that the head-on indicator is found to be a normally distributed random parameter, suggesting the estimations of the head-on collisions vary across the passenger vehicle–truck crashes (Figure 3). This heterogeneity may be due to several unobserved factors that are not accessible to the research team. The marginal effects of head-on collisions also show there is a 22% decreased possibility of sustaining no apparent/no injury, a 20.1% increased possibility of sustaining a minor injury, and a 2% increased possibility of suffering a serious/fatal injury. Likewise, a higher propensity of severe injury outcomes is also associated with greater numbers of injuries. 

## 5. Limitation

The study investigates the associations between unsafe driver actions and injury outcomes in passenger vehicle–truck collisions by using the real-world police-reported crashes, which has many intrinsic limitations [29,39]. For example, the driver actions reported in police crash reports were assumed to be accurate, and the intentional and unintentional driver actions were classified according to the description of police officers. However, it is often quite difficult for police officers to reconstruct what happened in a collision after the fact. Thus, the objectivity of the classification of intentional and unintentional actions might be a limitation of the study, as it is guessed by police who were not on the scene. Moreover, there are also some important variables that are not available in the databases, which, if incorporated, could better improve the estimation results. Such limitations will be solved in the near future by examining other high–quality crash datasets. 

## 6. Conclusions

This study contributes by quantifying associations of intentional and unintentional improper actions of truck and passenger vehicle drivers and driver condition with injury severity outcomes in passenger vehicle–truck collisions. The study explicitly accounts for unobserved heterogeneity and finds that some of the correlates have both positive and negative associations with injury severity. Rigorous fixed- and random-parameter ordered probit models are estimated using 2013 state-wide passenger vehicle–truck collision data from the Commonwealth of Virginia. Significant efforts went into processing the raw data and linking different databases for collecting important information on the crash, vehicle, and driver-related factors. The model results showed that compared with fixed-parameter and random-effects ordered probit models, the random-parameter model provides a superior fit to data at hand and provides fuller information regarding the relationships between key factors and the most severe injury outcomes. 

Compared with truck occupants, passenger vehicle occupants are six times more likely to sustain minor injuries and ten times more likely to suffer serious/fatal injuries. All else being equal, intentional improper actions and unintentional improper actions of the passenger vehicle drivers are both positively associated with higher injury severity, while the unintentional actions of the truck drivers are negatively associated with injury severity in passenger vehicle–truck crashes. Passenger vehicle driver-related factors such as driving under the influence of alcohol and fatigue are associated with higher injury outcomes. Importantly, compared with other times of day, passenger vehicle–truck collisions occurring late at night and in early mornings (01:00 and 08:00) are associated with higher injury severity in such collisions. 

From a behavioral perspective, a specific taxonomy of driver errors, whether intentional or unintentional, should be targeted given that passenger vehicle driver errors are significantly associated with higher injury outcomes. The safety literature shows numerous examples where intentional errors are associated with higher injury outcomes. However, in the case of passenger vehicle–truck collisions, unintentional errors are also of great concern. Driver awareness and training programs such as educating passenger vehicle drivers about driving carefully in the vicinity of trucks may also target driver errors that increase injury severity. Regarding driver behaviors and conditions, driving while fatigued or falling asleep and collisions occurring late at night and in early mornings are high-risk factors associated with injury severity. Countermeasures (e.g., regular sleep patterns) should be considered to minimize such risk factors. Finally, from a methodological standpoint, the study results imply that addressing unobserved heterogeneity is important in injury analysis of such collisions. Ignoring unobserved heterogeneity can mask important information embedded in data, which may affect the quantification of effects of risk factors and hence the development of appropriate strategies. Due to the limitations of this study, further work will be performed to examine more comprehensive crash databases in order to validate the findings by exploring the effects of underreporting issues of data quality. 

## Figures and Tables

**Table 1:** Combinations of different driver actions.

Possible Combinations	Truck Driver	Passenger Vehicle Driver
1	I	No
2	U	No
3	No	I
4	No	U
5	I	I
6	U	U
7	I	U
8	U	I
9	No	No

Note: “I” stands for an intentional improper action; “U” stands for an unintentional improper action; “No” stands for no improper action.

**Table 2:** Descriptive statistics of key variables.

Variable	Description	Mean	SD	VIF
**Driver actions**				
Action 1 indicator	1 if passenger vehicle driver undertook no improper action while truck driver undertook intentional improper action, 0 otherwise	0.375	0.484	2.487
Action 2 indicator	1 if passenger vehicle driver undertook no improper action while truck driver undertook unintentional improper action, 0 otherwise	0.079	0.270	1.263
Action 3 indicator	1 if passenger vehicle driver undertook intentional improper action while truck driver undertook no improper action, 0 otherwise	0.413	0.492	2.507
Action 4 indicator	1 if passenger vehicle driver undertook unintentional improper action while truck driver undertook no improper action,0 otherwise	0.133	0.340	1.640
**Collision type**				
Angle	1 If collision type is angle, 0 otherwise	0.298	0.457	1.290
Head-on	1 if collision type is head-on, 0 otherwise	0.017	0.130	1.060
Sideswipe-same direction	1 If collision type is sideswipe-same direction, 0 otherwise	0.306	0.461	1.350
Injury count	Total number of injuries in a collision	0.388	0.709	1.050
**Roadway Type**				
Interstate (Base)	1 if roadway type is interstate, 0 otherwise	0.409	0.492	3.640
principal arterial	1 if roadway type is principal arterial, 0 otherwise	0.269	0.443	3.060
minor arterial	1 if roadway type is minor arterial, 0 otherwise	0.186	0.389	2.543
collector	1 if roadway type is collector, 0 otherwise	0.101	0.301	1.835
local	1 if roadway type is local, 0 otherwise	0.035	0.184	1.168
**Driver condition**				
Drinking and driving (pass. veh. driver)	1 if the passenger vehicle driver is drunk, 0 otherwise	0.023	0.148	1.030
Fatigued driving (pass. veh. driver)	1 if the passenger vehicle driver is fatigued or asleep, 0 otherwise	0.019	0.135	1.060
**Demographics**				
Age (pass. veh. driver)	1 if the passenger vehicle driver is 20–29 years old, 0 otherwise	0.241	0.428	1.020
**Time of Day**				
Late night/early morning	1 if a crash happened between 01:00 and 08:00, 0 otherwise	0.207	0.405	1.010

Notes: Intentional actions refer to actions including speeding, wrong places, no right-of-way, following too close, disregard of officers, signals, or signs, and so forth. Unintentional actions refer to actions including avoiding animals or objects, failing to maintain proper control, other improper actions, and so forth. Pass. veh. refers to the abbreviation of the passenger vehicle.

**Table 3:** Modeling results for fixed- and random-parameter ordered probit models.

	Fixed-Parameter Model		Random-Parameter Model	
Variable	β	*t*-Stats	β	*t*-Stats
**Driver actions**				
Action 1 (Base)				
Action 2	−0.167	−1.43	−0.391 ***	−2.93
standard deviation	---	---	0.658 ***	5.45
Action 3	0.133 **	2.07	0.150 **	2.09
Action 4	0.315 ***	3.72	0.381 ***	4.32
**Collision type**				
Angle	0.160 **	2.50	0.178 ***	2.63
Head-on	0.793 ***	4.62	0.856 ***	4.39
standard deviation	---	---	0.932 ***	4.62
Sideswipe-same direction	−0.262 ***	−3.46	−0.273 ***	−3.10
Injury count	1.310 ***	36.54	1.571 ***	43.80
**Roadway Type**				
Interstate (Base)				
principal arterial	−0.142 **	−2.02	−0.433 ***	−5.40
standard deviation	---	---	0.849 ***	15.02
minor arterial	−0.253 ***	−3.17	−0.640 ***	−6.78
standard deviation	---	---	1.004 ***	14.43
collector	−0.227 **	−2.29	−0.297 ***	−2.94
local	−0.531 ***	−3.11	−0.639 ***	−3.94
**Driver condition**				
Drinking and driving (pass. veh. driver)	0.533 ***	3.54	0.581 ***	3.67
Fatigued driving (pass. veh. driver)	0.299 *	1.75	0.335 *	1.86
**Demographics**				
Age (pass. veh. driver)	−0.158 **	−2.39	−0.187 ***	−2.62
standard deviation	---	---	0.232 ***	3.77
**Time of Day**				
Late night/early morning	0.196 ***	3.06	0.270 ***	3.82
μ(1)	1.262 ***	26.71	1.518 ***	29.25
**Number of observations**	3774		3774	
Log-likelihood with constant only	−2408.439		−2408.439	
Log-likelihood at convergence	−1494.187		−1472.797	
Likelihood Ratio Test	Chi–square = 42.780; *p*–value = 0.005			

Notes: ***, **, * represents significance at 1%, 5%, and 10% level. μ(1) represents estimable threshold parameters that define the most severe injury outcomes of passenger vehicle–truck collisions.

**Table 4:** Marginal effects (fixed- and random-parameter ordered probit models).

	Fixed-Parameter Model			Random-Parameter Model		
Variables	No Apparent/No Injury	Minor Injury	Serious/Fatal Injury	No Apparent/No Injury	Minor Injury	Serious/Fatal Injury
**Driver actions**						
Action 2 indicator	0.032	−0.029	−0.003	0.050	−0.049	−0.002
Action 3 indicator	−0.028	0.025	0.003	−0.025	0.024	0.001
Action 4 indicator	−0.074	0.065	0.009	−0.074	0.070	0.004
**Collision type**						
Angle	−0.035	0.031	0.004	−0.030	0.029	0.001
Head-on	−0.234	0.190	0.044	−0.220	0.201	0.020
Sideswipe-same direction	0.051	−0.046	−0.005	0.041	−0.039	−0.002
Injury count	−0.272	0.243	0.029	−0.253	0.243	0.010
**Roadway Type**						
principal arterial	0.028	−0.025	−0.003	0.061	−0.059	−0.002
minor arterial	0.048	−0.043	−0.005	0.079	−0.076	−0.003
collector	0.042	−0.038	−0.004	0.041	−0.039	−0.001
local	0.081	−0.075	−0.007	0.068	−0.066	−0.002
**Driver condition**						
Drinking and driving (pass. veh. driver)	−0.143	0.121	0.022	−0.131	0.122	0.009
Fatigued driving (pass. veh. driver)	−0.072	0.063	0.009	−0.066	0.063	0.004
**Demographics**						
Age (pass. veh. driver)	0.031	−0.028	−0.003	0.028	−0.027	−0.001
**Time of Day**						
Late night/early morning	−0.043	0.038	0.005	−0.048	0.046	0.002
